# GDF11 improves tubular regeneration after acute kidney injury in elderly mice

**DOI:** 10.1038/srep34624

**Published:** 2016-10-05

**Authors:** Ying Zhang, Qinggang Li, Dong Liu, Qi Huang, Guangyan Cai, Shaoyuan Cui, Xuefeng Sun, Xiangmei Chen

**Affiliations:** 1 Department of Nephrology, Chinese PLA General Hospital, Chinese PLA Institute of Nephrology, State Key Laboratory of Kidney Diseases, National Clinical Research Center for Kidney Diseases, Beijing, 100853, China; 2 Medical College, Nankai University, Tianjin, 300071, China; 3 Department of Nephrology, Chinese PLA Air Force General Hospital, Beijing, 100142, China

## Abstract

The GDF11 expression pattern and its effect on organ regeneration after acute injury in the elderly population are highly controversial topics. In our study, GDF11/8 expression increased after kidney ischemia–reperfusion injury (IRI), and the relatively lower level of GDF11/8 in the kidneys of aged mice was associated with a loss of proliferative capacity and a decline in renal repair, compared to young mice. *In vivo*, GDF11 supplementation in aged mice increased vimentin and Pax2 expression in the kidneys as well as the percentage of 5-ethynyl-2′-deoxyuridine (EdU)-positive proximal tubular epithelial cells. GDF11 improved the renal repair, recovery of renal function, and survival of elderly mice at 72 h after IRI. Moreover, the addition of recombinant GDF11 to primary renal epithelial cells increased proliferation, migration, and dedifferentiation by upregulating the ERK1/2 pathway *in vitro*. Our study indicates that GDF11/8 in the kidney decreases with age and that GDF11 can increase tubular cell dedifferentiation and proliferation as well as improve tubular regeneration after acute kidney injury (AKI) in old mice.

Acute kidney injury (AKI) is associated with significantly increased morbidity and mortality and predisposes patients to chronic kidney disease. The incidence of AKI has steadily increased in recent years, and this increase is strongly associated with the advancing age of our population^1^. In response to acute injury, the kidney normally has the ability to initiate a burst of cellular proliferation to repopulate and restore injured tubules, which can lead to full functional recovery and partial kidney repair. However, the proliferative burst appears to decline with age^2^,^3^, leading to a decrease in the recovery of renal function in the elderly. Patients older than 65 years old have a 28% higher risk of failing to recover renal function completely after surviving an episode of AKI^4^. Despite the recent significant progress in our understanding of dedifferentiated renal tubular cells as the primary cells that proliferate and repair injured tubules^5^,^6^, the mechanisms of tubular cell dedifferentiation and the role of age in this process have not been elucidated.

Growth differentiation factor (GDF) 11 is a key factor in embryonic development that maintains stemness of stem cells and inhibits their differentiation. Like other transforming growth factor (TGF)-β ligands, GDF11 is synthesized as a precursor molecule consisting of a signal peptide, an N-terminal prodomain, and a C-terminal mature domain^7^. The prodomain of GDF11 is cleaved by proprotein convertases (PC) 5/6 at the canonical S1 site containing the R293xxR296 motif^8^. Then, the severed prodomain remains noncovalently associated with the mature region of the molecule in the latent complex and is secreted from the cell. Finally, the GDF11 prodomain is cleaved by the bone morphogenetic protein-1/tolloid (BMP-1/TLD) family of extracellular metalloproteases, and GDF11 is activated^9^. As it has been reported that PC5/6-deficient embryos exhibit GDF11-related phenotypes such as altered anteroposterior patterning with extra vertebrae, a lack of a tail, and renal agenesis, PC5/6 is an important protease that regulates GDF11^8^. Thus, GDF11 activation is regulated by two proteases after it is translated, and the mature peptide dimer is its functional form.

In recent years, the changing level of GDF11 in circulation with age as well as its relative antihypertrophic effect and prorepair effect in elderly mice are highly controversial, leaving unsolved questions^10^,^11^,^12^,^13^,^14^. However, this does not imply the autocrine and paracrine GDF11 is without effect. GDF11 is highly expressed in the metanephric mesenchyme and ureteric bud, and it is necessary in metanephric development^8^,^15^. The GDF11 mRNA level is the highest in the kidney, and GDF11 expression in cancerous kidney tissue is much higher than that in normal kidney tissue^16^. These findings indicate that GDF11 is tightly associated with the proliferative state of the kidney. However, whether GDF11 mediates the proliferative burst and tubular cell dedifferentiation in the elderly with AKI is unknown.

Ischemic insult is the main cause of AKI in the clinic. In this study, we aimed to demonstrate the relationships between GDF11/8 expression, the proliferative response after kidney ischemia–reperfusion injury (IRI), and renal repair. In addition, the *in vivo* effects of recombinant GDF (rGDF)11 supplementation in aged mice on epithelial cell dedifferentiation and proliferation during the repair phase were assessed. Furthermore, the *in vitro* effects of the addition of rGDF11 to primary renal epithelial cells on proliferation, migration, and dedifferentiation as well as the ERK1/2 signaling pathway were determined.

## Materials and Methods

### Mice

All experiments were performed using C57Bl/6 mice at ages of 3 months (young group) or 23–24 months (old group). Mice were purchased from the Si Bei Fu Laboratory Animal Company and housed under specific pathogen-free conditions in the Experimental Animal Center of the Academy of Military Medical Sciences. The experimental protocol was carried out in accordance with the approved guidelines of the Institutional Animal Care and Use Committee at the Chinese PLA General Hospital. The details of the IRI experiment and tissue preparation are provided in the following section. To further explore the role of GDF11 on IRI in old mice, we restored the GDF11 levels in the kidneys of old mice by a daily intraperitoneal (i.p.) injection of rGDF11 (Pepro Tech; 0.3 mg/kg/d in phosphate-buffered saline, PBS) from 48 h before IRI to 48 h after IRI, and the control group was given an equal volume of vehicle (PBS containing 0.1% bovine serum albumin (BSA) and 4 mM HCl). At the scheduled day of sacrifice, the mice were anesthetized by an i.p. injection of pentobarbital sodium. Blood was collected from the abdominal vein, and the kidneys were collected after perfusion with saline.

### IRI experiments

Before starting the experiment, all surgical tools were sterilized by autoclaving and aseptic towels and sterile materials were prepared. IRI studies were performed under standardized conditions on a super clean bench, as described elsewhere^17^. Briefly, after the mice were anesthetized by an i.p. injection of pentobarbital sodium (40 mg/kg for old mice and 60 mg/kg for young mice) and sterilized by iodophor, a midline abdominal incision was made, and bilateral renal pedicles were clipped at specific times (28, 35, 40 min for young mice and 28 and 35 min for old mice) using microaneurysm clamps (Harvard Apparatus 728816). After removal of the clamps, kidney reperfusion was visually confirmed by the color change from dark purple to pink. The incision was then closed, and the animal was injected with 1.5 mL of saline subcutaneously and allowed to recover. During the ischemic period, the body temperature was maintained at approximately 37 °C using a temperature-controlled heating system. Sham-operated mice received identical surgical procedures except that clamps were not applied.

### Histopathological examination for acute tubular necrosis scores

Paraffin sections were stained with periodic acid–Schiff reagent by standard protocols. Histological examinations were performed independently in a blind fashion by two observers. The severity of acute tubular necrosis was quantified by counting the percentage of tubules in the corticomedullary junction that displayed cell necrosis, loss of brush borders, cast formation, and tubular dilatation as follows: 0 = none, 1 = ≤10%; 2 = 11–25%; 3 = 26–45%; 4 = 46–75%; and 5 = >76%. Approximately 40 high-power fields (HPFs, 400×) per individual mouse (10 HPFs per slide, four slides per animal) were evaluated; n = 6 in each group.

### 5-Ethynyl-2′-deoxyuridine incorporation

5-Ethynyl-2′-deoxyuridine (EdU) staining was performed using the protocol provided by the Click-iT® EdU Imaging Kit (Life Technologies). For the *in vivo* experiments, the mice were intraperitoneally injected with 5 μg/g EdU after IRI. For the *in vitro* experiments, cells on Matrigel-coated coverslips were incubated with 5 μM EdU for 24 h. The slides with frozen kidney sections or cells were fixed with 4% (vol/vol) paraformaldehyde for 15 min at room temperature, permeabilized with 0.5% Triton X-100 in PBS for 5 min, rinsed three times for 10 min each, and incubated with Click-iT® reaction cocktail for 30 min at room temperature. Finally, the slides were incubated with FITC-conjugated anti-lotus tetragonolobus lectin (LTL) (FL-1321; Vector Labs; 1:1,000) for 1 h at room temperature. Nuclear counterstaining was performed using DAPI, and then the samples were mounted with Prolong-Gold (Invitrogen). Images were obtained by confocal (Nikon C1 Eclipse; Nikon) or standard (Nikon Eclipse 90i; Nikon) microscopy. The percentage of EdU-positive tubular cells was quantified per HPF (200×). Approximately 20 HPFs per individual mouse (5 HPFs per slide, four slides per animal) were evaluated; n = 6–8 in each group. The percentage of EdU-positive human primary renal proximal tubular epithelial cells (hPTCs) was quantified per low-power field (LPF, 40×). Approximately 15 LPFs per group from three independent experiments were evaluated.

### Immunofluorescence staining

Immunofluorescence protocols and antibodies are detailed below. Frozen sections were used for immunofluorescence staining. Primary cultured tubular epithelial cells on Matrigel-coated coverslips and kidney sections were permeabilized with 0.2% Triton X-100 and fixed with 4% (vol/vol) paraformaldehyde for 5 min. Samples were blocked with 5% (vol/vol) normal goat serum in PBS and incubated with primary antibodies including FITC-conjugated anti-lotus LTL (FL-1321; Vector Labs; 1:1,000), goat anti-α-smooth muscle actin (SMA) (1:1,000, Abcam, ab21027), goat anti-kidney injury molecule-1 (Kim-1) (AF1817; R&D Systems; 1:500), rabbit anti-Pax2 (ab92547; Abcam; 1:500), and rabbit anti-vimentin (#5741; CST; 1:500). Secondary antibodies were either FITC- or Cy3-conjugated (Jackson ImmunoResearch) and incubated for 1 h. Nuclear counterstaining was performed using DAPI (Invitrogen). Images were obtained by confocal (Nikon C1 Eclipse; Nikon) or standard (Nikon Eclipse 90i; Nikon) microscopy.

### Western blot

Protein samples were electrophoresed in a 10% or 12% sodium dodecyl sulfate–polyacrylamide gel and transferred to nitrocellulose membranes (Bio-Rad, Hercules, CA, USA). Then, the membrane was incubated with antibodies against GDF11 (Abcam, ab124721; 1:1,000 or Biorbyt, orb101175; 1:500, which detects mature GDF11 at 12.5 kDa), Pax2 (Abcam, ab92547; 1:1,000), vimentin (CST, #5741; 1:1,000), Kim1 (R&D Systems, AF1817; 1:1,000), E-cadherin (Abcam, ab15148; 1:1,000), α-SMA (Abcam, ab21027; 1:1,000), ERK1/2 (CST, #9102; 1:1,000), p-ERK1/2 (CST, #9101; 1:1,000), cyclin D1 (Abcam, ab7958; 1:1,000), and c-Myc (Abcam, ab32072; 1:1,000) and washed with Tris-buffered saline containing 0.1% Tween 20. The membrane was incubated with the secondary immunoglobulins conjugated with horseradish peroxidase. The bound antibodies were detected with detection reagent (Pierce, Rockford, IL, USA). Quantification of the band intensity was accomplished with NIH Image J software.

### RNA extraction and real-time quantitative polymerase chain reaction (PCR)

Total RNA was isolated using TRIzol (Invitrogen, Carlsbad, CA, USA). The high capacity cDNA Reverse Transcription Kit (Applied Biosystems, Foster City, CA, USA) and a GeneAmp® PCR System 9700 (Applied Biosystems) were used to generate cDNA. Gene expression analysis was determined by quantitative real-time PCR using Taqman Master mix and specific probe and primer compounds for GDF11 (Mm01159973_m1, Applied Biosystems) and GDF8 (Mm 01254559_m1, Applied Biosystems). PCR was performed on a 7500 Real-time PCR System (Applied Biosystems), and the results were analyzed using the 2-ΔΔCT method with normalization against glyceraldehyde 3-phosphate dehydrogenase expression (n = 6 for each group).

### Cell culture and treatment

American type culture collection (ATCC)® Normal Human Primary Renal Proximal Tubule Epithelial Cells (hPTC) were grown in Renal Epithelial Cell Basal Media (PCS-400-030,ATCC) supplemented with Renal Epithelial Cell Growth Kit components (PCS-400-040). The final medium included fetal bovine serum (0.5%), triiodothyronine (10 nM), recombinant human epidermal growth factor (10 ng/mL), hydrocortisone hemisuccinate (100 ng/mL), recombinant human insulin (5 mg/mL), epinephrine (1.0 mM), transferrin (5 mg/mL), and L-alanyl-L-glutamine (2.4 mM). The experimental group was treated with 80 ng/mL rGDF11 (Perprotec) in 0.1% BSA, and the control group was administered an equal volume of 0.1% BSA. To measure migration, hPTCs were grown to confluence, and monolayers were wounded with a rubber policeman to produce a linear 4-mm swipe. After one wash with PBS, cells were cultured in medium in the presence of 80 ng/mL rGDF11 or 0.1% BSA. After 36 h, cell migration was determined using a microscope and camera, and the wound area was calculated using NIH Image J software. To measure hPTC proliferation, 2,500 cells/well were seeded into 96-well plates, and the relative cell viability at each experimental time was determined by using a cell counting kit (DojinDo).

### Phospho-protein profiling by a phospho-antibody array

The experiment was performed by Wayen Biotechnologies (Shanghai), Inc. Cell lysates obtained from cells treated with rGDF11 or 0.1% BSA were applied to the TGF-β Pathway Phosphorylation Antibody Array that was designed and manufactured by Full Moon Biosystems Inc. The array contained 176 antibodies, each of which had 6 replicates that were printed on standard-size coated glass microscope slides. In brief, 25 μg of cell lysate in 67 μL of reaction mixture was labeled with 3 μL of biotin in 10 μg/μL *N,N*-dimethyformamide. The resulting biotin-labeled proteins were diluted with 6 mL of Coupling Solution (Full Moon Biosystems Inc.) before being applied to the array for conjugation. The antibody microarray was first blocked with blocking solution for 30 min at room temperature, rinsed with Milli-Q-grade water for 3 min, and dried with compressed nitrogen, followed by incubation with the biotin-labeled cell lysates at 4 °C overnight. The array slides were washed three times with 60 mL of 1 × Wash Solution (Full Moon Biosystems Inc.) for 10 min each. The conjugated labeled proteins were detected using Cy3-conjugated streptavidin. For each antibody, the following phosphorylation ratio was computed (phosphorylated and matching unphosphorylated values are denoted by phospho and unphospho in both the control data and experimental data):


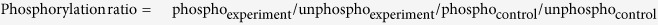


The mean of the phosphorylation ratio was obtained from two independent experiments.

### Statistical analysis

Data are expressed as means ± standard deviation (SD). Statistical significance was assessed by the Student’s t-test for two groups, one-way analysis of variance (ANOVA) for more than two groups, and two-way ANOVA for factorial designed groups. Cumulative survival was analyzed by the log rank test (Kaplan–Meier). Differences were considered statistically significant at *P < *0.05.

## Results

### Impaired kidney recovery and higher mortality in old mice at 72 h after IRI were associated with a decline in the dedifferentiation and proliferation of tubular cells

**Figure 1:**
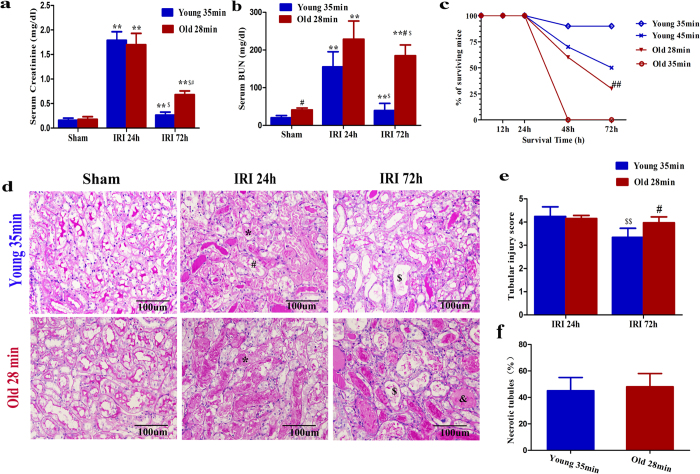
Survival and renal function of young mice that underwent 35 min of IRI and of old mice that underwent 28 min of IRI. (**a,b**) Serum creatinine (**a**) and BUN values (**b**) at 24 h and 72 h for young mice that underwent 35 min of IRI and for old mice that underwent 28 min of IRI. (**c**) Survival curves for young mice after 35 min or 45 min of IRI and old mice after 28 min or 35 min of IRI. (**d,e**) Renal histology (**d**) and tubular injury scores (**e**) at 24 h and 72 h of young mice that underwent 35 min of IRI and old mice that underwent 28 min of IRI. (**f**) The percentage of necrotic tubules at 24 h of young mice that underwent 35 min of IRI and old mice that underwent 28 min of IRI. For serum creatinine (**a**) and BUN (**b**), values are means ± SD, n = 6–8 in each group. ^**^P < 0.01 vs. sham, ^#^*P* < 0.05 vs. young mice with 35 min of IRI, ^$^*P* < 0.05 vs. 24 h after IRI. For survival curves (**c**), ^##^*P* < 0.01 vs. young mice with 35 min of IRI by log-rank, n = 10 in young mice, 35 min of IRI, n = 20 in old mice, 28 min of IRI. For renal histology (**d**), original magnification, 400 × . *Indicates necrotic tubules, ^#^indicates loss of brush borders ^&^indicates tubule dilatation, and ^$^indicates cast formation. For tubular injury scores (**e**) and necrotic tubules (**f**), values are means ± SD. Approximately 40 HPFs (magnification, 400 × ) per individual mouse (10 HPFs per slide, four slides per animal) were evaluated; n = 6 in each group. ^#^*P* < 0.05 vs. young mice with 35 min of IRI, ^$$^*P* < 0.01 vs. 24 h after IRI.

**Figure 2:**
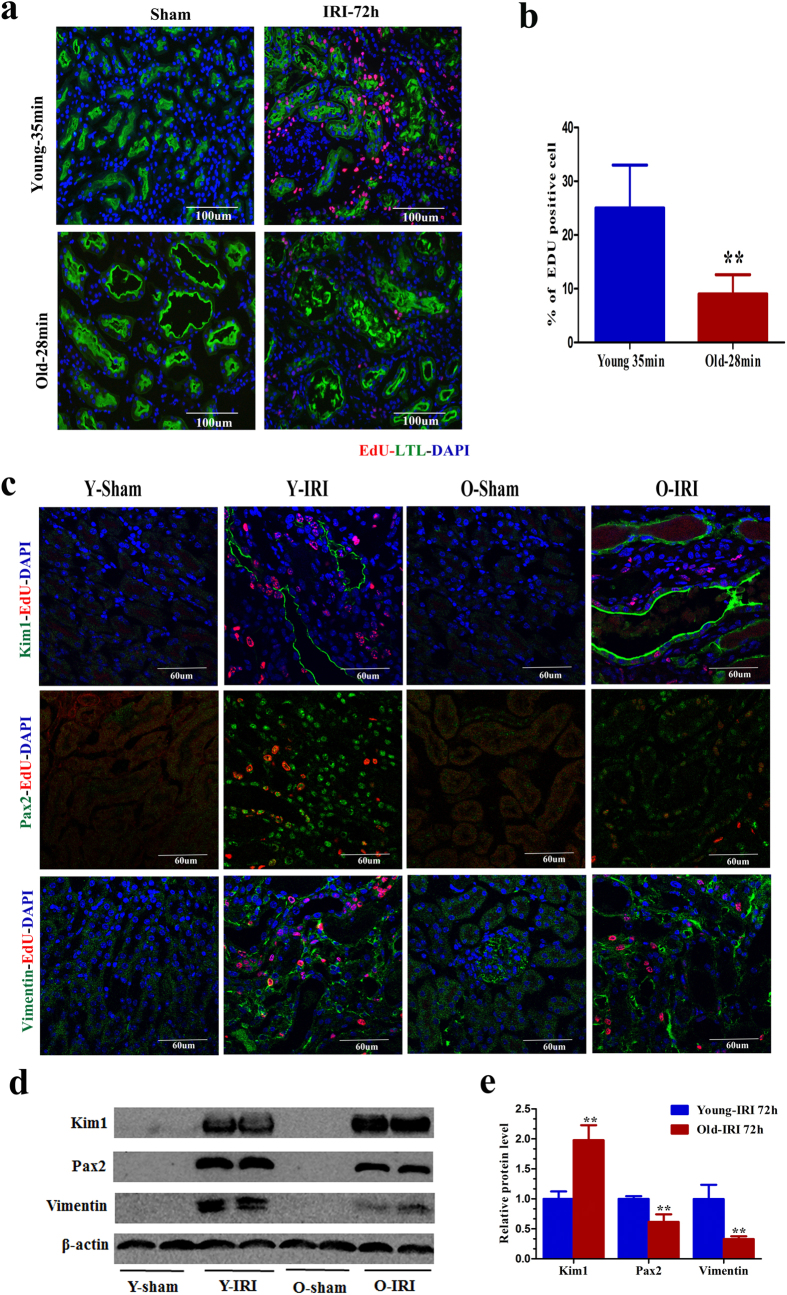
Tubular cell proliferation and dedifferentiation declined in old mice at 72 h after 28 min of IRI compared with young mice after 35 min of IRI. (**a,b**) The percentage of EdU-positive tubular cells was smaller in the kidneys of old mice at 72 h after 28 min of IRI compared with young mice after 35 min of IRI. The photomicrographs of the inner cortex and outer medulla are shown with a magnification of 200× (**a**). (**b**) The percentage of EdU-positive tubular cells/high power field in the inner cortex and outer medulla. Approximately 20 HPFs (magnification, 200 ×) per individual mouse (5 HPFs per slide, four slides per animal) were evaluated. Values are means ± SD, n = 6–8 in each group, ^**^P < 0.01 vs. young mice with 35 min of IRI. (**c**) Expression of the proximal tubular cell injury marker Kim1 and the dedifferentiation markers Pax2 and vimentin at 72 h in sham kidneys, young kidneys that underwent 35 min of IRI and old kidneys that underwent 28 min of IRI (original magnification, 600×). (**d**) Western blot for Kim1, Pax2, and vimentin expression in whole-kidney lysates from young mice that underwent 35 min of IRI and old mice that underwent 28 min of IRI (each lane represents a separate mouse). (**e**) Relative quantification of the Kim1, Pax2, and vimentin expression in (**d**), normalized to actin. Values are means ± SD, n = 4 in each group, ^**^P < 0.01 vs. young mice with 35 min of IRI.

We first tried to distinguish the kidney function changes and histological injuries between old and young mice during AKI. Serum creatinine, blood urea nitrogen (BUN), and histological injuries of young mice that underwent 28 min, 35 min, and 40 min of bilateral IRI and old mice that underwent 28 min and 35 min of bilateral IRI were analyzed (Supplementary Tables 1–3). Old mice had a larger increase in serum creatinine and BUN levels at 24 h after IRI than young mice that underwent the same length of ischemia (Supplementary Tables 1 and 2). Old mice had a much larger percentage of necrotic tubules at 24 h after IRI than young mice that underwent the same length of ischemia (Supplementary Table 3). It was demonstrated that the old mice received a much greater kidney insult relative to the young animals with the same length of ischemia. Young mice with 35 min of IRI and old mice with 28 min of IRI had similar increases in serum BUN and creatinine levels (Fig. 1a,b) as well as comparable tubular injury and necrotic tubules at 24 h after IRI (Fig. 1d–f). These findings suggested that old mice with 28 min of IRI and young mice with 35 min of IRI have equivalent kidney injury at 24 h after IRI, the time point of most serious injury. To avoid the effect of the initial injury on the later kidney function recovery and tubule regeneration, the study performed young mice with 35 min of IRI and old mice with 28 min of IRI in the next study. Noncomparable changes in serum BUN and creatinine levels were observed at 72 h after IRI, when young mice exhibited a steady decrease of BUN and creatinine levels but a lack of functional recovery was only observed in the old mice (Fig. 1a,b). The kidney function changes in the old and young mice and the distinction between the old and young mice were confirmed by the tubular injury scores (Fig. 1d,e). A decline of functional recovery and organ repair in the old mice led to higher mortality at IRI 72 h, compared to young mice with 35 min of IRI (Fig. 1c). Kidneys from young mice had significantly higher rates of proximal tubular cell proliferation at 72 h after 35 min of IRI than did kidneys from old mice with 28 min of IRI, as demonstrated by the percentage of EdU-positive proximal tubular cells (Fig. 2a,b). Because dedifferentiated tubular cells are the primary cells to restore injured tubules, we next tested whether tubular cell dedifferentiation changed in the old kidney. Kim1, a marker of proximal tubular injury, and vimentin and Pax2, markers of tubular cell dedifferentiation, were not detected in sham group kidneys (Fig. 2c). However, these proteins were observed in the kidneys of young mice that underwent 35 min of IRI and old mice that underwent 28 min of IRI (Fig. 2c). Although Kim1 expression was higher in the kidneys of old mice at 72 h after 28 min of IRI, vimentin and Pax2 expression was lower, compared with the kidneys of young mice (Fig. 2d,e). Our results suggested that the dedifferentiation and proliferation of injured tubular cells were decreased in old mice at 72 h after IRI, which might result in a reduced recovery of renal function and repair in old mice.

### GDF11/8 localization in the kidney and expression pattern during AKI

**Figure 3:**
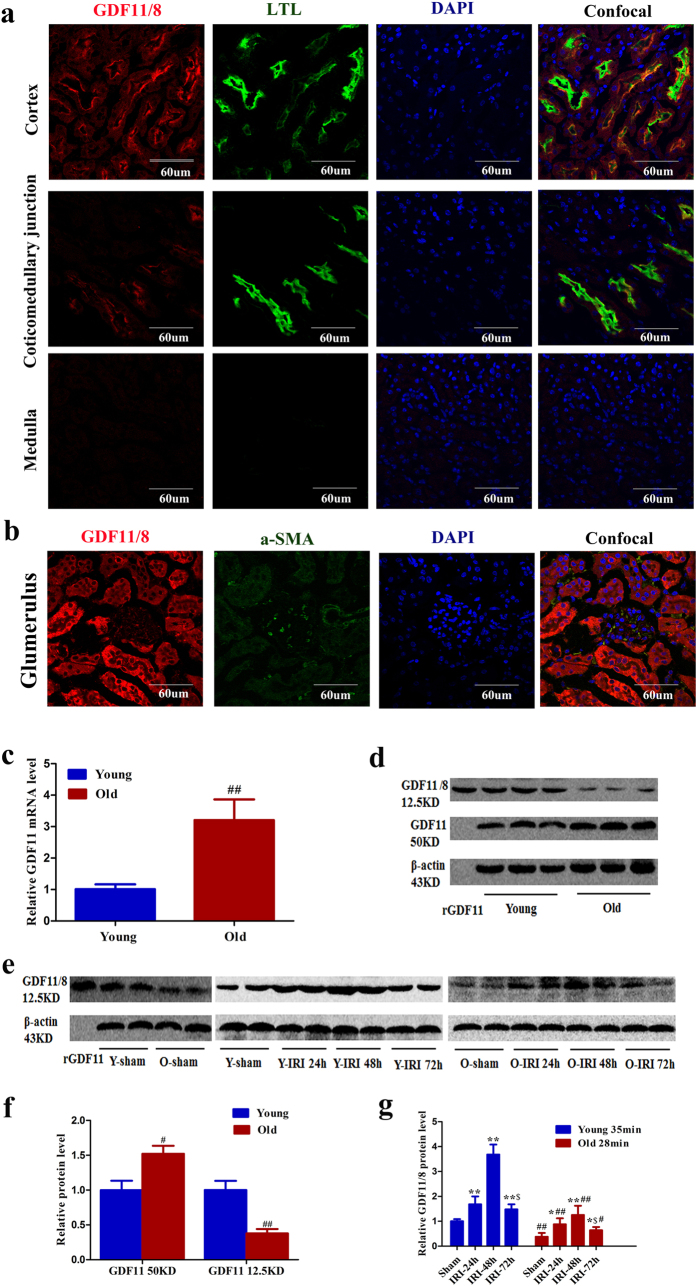
GDF11/8 localization in the kidney and expression pattern during AKI. (**a,b**) Immunofluorescence was performed using a GDF11 antibody (ab124721) on normal kidney sections of 3-month-old C57Bl/6 mice. GDF11 was mainly expressed in the proximal tubular cells (confocal with LTL) and slightly expressed in the mesangial cells (confocal with α-SMA). Magnification, 600×. (**c,d,f**) GDF11 mRNA (**c**) and protein (**d,e**) expression in the kidneys of 3-month-old and 24-month-old C57Bl/6 mice (each lane represents a separate mouse). (**e,g**) The mature GDF11 peptide increased in the kidneys of 3-month-old and 24-month-old C57Bl/6 mice that underwent IRI (each lane represents a separate mouse). For GDF11 mRNA (**c**), data are means ± SD, n = 6 in each group, ^##^*P* < 0.01 vs. young mice. For GDF11 protein in the kidneys of young and old mice (**f**), data are means ± SD, n = 3 in each group, ^#^*P* < 0.05 vs. young mice, ^##^*P* < 0.01 vs. young mice. For GDF11 protein in the kidneys of young and old mice that underwent IRI (**g**), data are means ± SD, n = 4 in each group, ^*^P < 0.05 vs. Sham, ^**^P < 0.01 vs. Sham, ^#^*P* < 0.05 vs. young mice with 35 min of IRI, ^##^*P* < 0.01 vs. young mice with 35 min of IRI, ^$^*P* < 0.05 vs. IRI at 48 h after IRI.

Because the mature peptides of GDF11 and GDF8 are highly homologous, most of the GDF11 antibody cross-reacts with GDF8. Unfortunately, the only GDF11-specific antibody (R&D Systems 743833) is not applicable for western blot as it does not detect denatured protein. In addition, it is from mouse, so it is insensitive to detect GDF8/11 in mouse tissue as reported by Egerman^12^. Therefore, GDF11 and GDF8 mRNA levels in the kidney were first examined by quantitative PCR. To detect the GDF8 mRNA level in the kidney, skeletal muscle was used as a positive control to be sure that the PCR system used was working well. The GDF11 mRNA levels in the muscle, spleen, and kidneys are shown in Supplementary Fig. 1a, and the GDF11 and GDF8 mRNA levels in the skeletal muscle are shown in Supplementary Fig. 1b. GDF8 mRNA was not detected by PCR, while the cycle threshold value of GDF11 was 21 in the kidney, and the same expression pattern was found in the spleen. Therefore, the endogenous GDF8 level was very low in the kidney and spleen, compared with the muscle. Although mature GDF11 and myostatin ligands share substantial sequence identity, their prodomains are only 52% identical. GDF11 and GDF8 precursors can be distinguished from each other due to different molecular weights: the former is 50 kDa, and the later 47 kDa. As GDF8 in the circulation might reach the kidney, little GDF8 might be detected by the ab124721 antibody. The GDF11 precursor at 50 kDa, the C-cleavage precursor product at 35 kDa, and the mature peptides of GDF11/8 (probably most of which was GDF11) at 12.5 kDa in the kidney were detected by western blot using the ab124721 antibody, as previously reported in PC12 cells by Ge^9^. In addition, the GDF11 precursor level was much higher than that of the GDF11/8 mature peptide (Supplementary Fig. 1d). GDF11/8 (most of which was the GDF11 precursor) was completely colocalized with proximal tubular epithelial cells in the cortex and coticomedullary junction and with the mesangial cell marker α-SMA in the glomerulus, as detected by immunofluorescence using the ab124721 antibody (Fig. 3a,b). The highest GDF11/8 (most of which was the GDF11 precursor) expression was detected in the proximal tubules, with lower levels in the mesangial cells (Fig. 3a,b). The GDF11 mRNA and precursor molecule levels were higher in the kidneys of old mice compared with young mice, while the mature molecule of GDF11/8 was lower in old mice (Fig. 3c,d,f). Next, we used the antibody orb101175 to detect mature GDF11/8 peptide. In the kidneys of young and old mice after IRI, the mature GDF11/8 (most of which was GDF11) molecule was upregulated at 24 h after IRI, reaching a maximum at 48 h after IRI and then decreasing at 72 h after IRI (Fig. 3e,g). The mature GDF11/8 expression was lower in old kidneys than in young kidneys at every time point (Fig. 3e,g). The GDF8 mRNA was not detected in the kidneys of young or old mice after IRI. In conclusion, the mature GDF11/8 (probably most of which was GDF11) peptide increased in response to tubular cell proliferation during IRI, and, in parallel with the decreased proliferation, their level was lower in old mice with IRI compared to young mice.

### Effect of GDF11 on renal repair, tubular cell dedifferentiation, and proliferation

**Figure 4:**
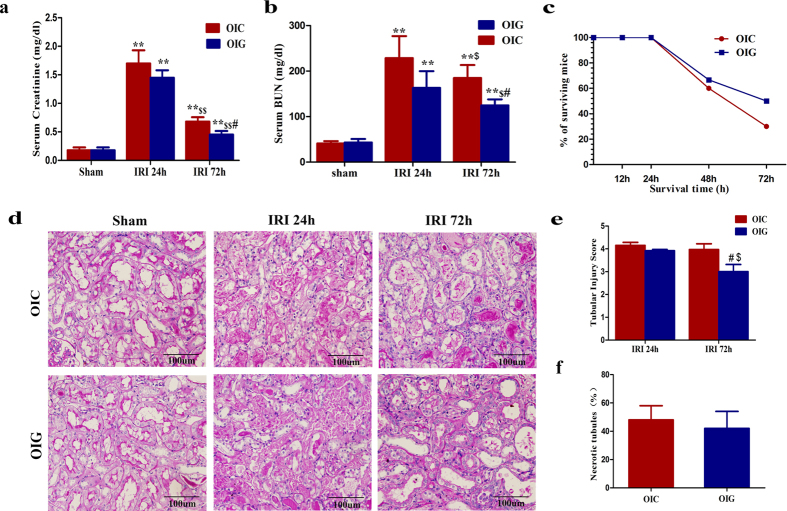
GDF11 increased the survival, renal function, and renal repair of old mice that underwent 28 min of IRI. (**a,b**) Serum creatinine (**a**) and BUN levels (**b**) at 24 h and 72 h for old mice that underwent 28 min of IRI in the control and GDF11 groups. (**c**) Survival curves at 72 h for old mice that underwent 28 min of IRI in the control and GDF11 groups. (**d,e**) Renal histology (**d**) and tubular injury scores (**e**) at 24 h and 72 h of old mice that underwent 28 min of IRI in the control and GDF11 groups (original magnification, 400×). (**f**) The percentage of necrotic tubules at 24 h of old mice that underwent 28 min of IRI in the control and GDF11 groups. For serum creatinine (**a**) and BUN (**b**), values are means ± SD, n = 6 in each group. OIC, old mice that underwent IRI in the control group; OIG, old mice that underwent IRI in the GDF11 group; ^**^P < 0.01 vs. sham, ^#^*P* < 0.05 vs. OIG, ^$^*P* < 0.05 vs. 24 h after IRI, ^$$^*P* < 0.01 vs. 24 h after IRI. For survival curves (**c**), OIC, old mice that underwent IRI in the control group; OIG, old mice that underwent IRI in the GDF11 group; *P* > 0.05 by the log-rank test, n = 20 in OIC, n = 12 in OIG. For tubular injury scores (**e**) and necrotic tubules (**f**), values are means ± SD. Approximately 40 HPFs (magnification, 400×) per individual mouse (10 HPFs per slide, four slides per animal) were evaluated. Values are means ± SD, n = 5–6 in each group. OIC, old mice that underwent IRI in the control group; OIG, old mice that underwent IRI in the GDF11 group; ^#^*P* < 0.05 vs. OIC, ^$^*P* < 0.05 vs. 24 h after IRI.

**Figure 5:**
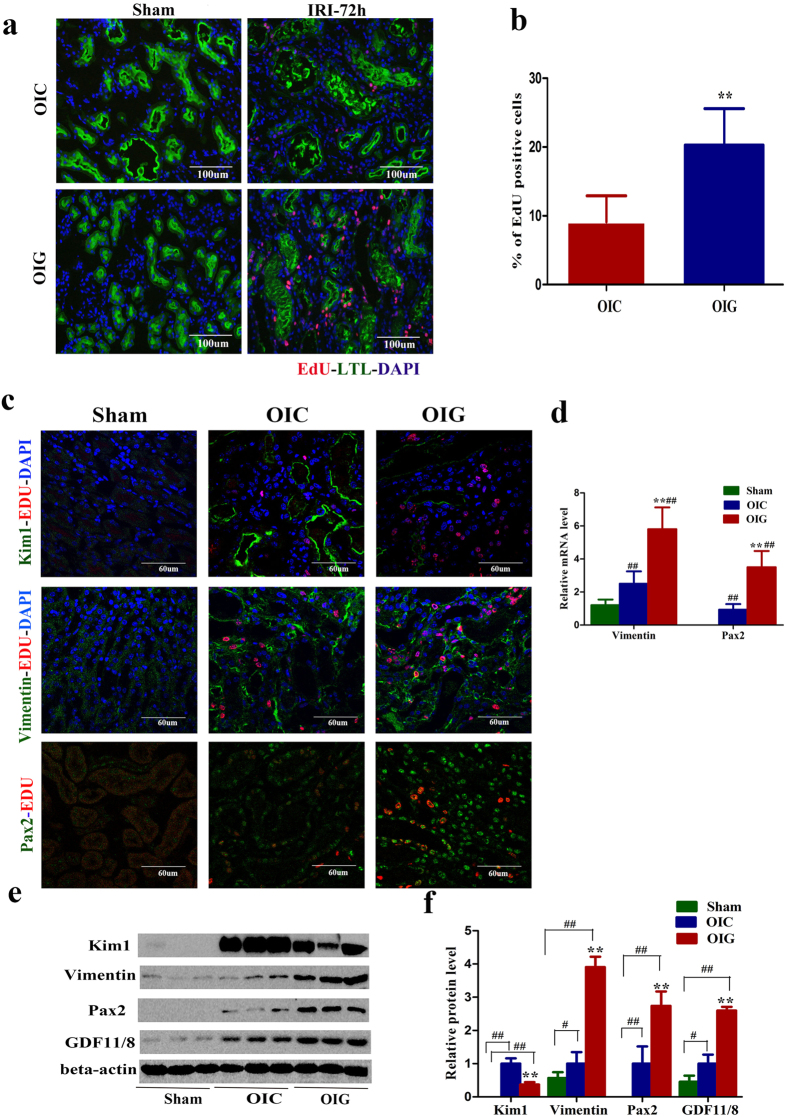
GDF11 enhanced tubular cell proliferation and dedifferentiation in old mice that underwent 28 min of IRI. (**a,b**) Tubular cell proliferation increased in old mice treated with GDF11 that underwent 28 min of IRI. Pictures are shown with 200 × magnification (**a**). (**b**) Percentage of EdU-positive tubular cells/HPF in the outer medulla and inner cortex. Approximately 20 HPFs (magnification, 200×) per individual mouse (5 HPFs per slide, four slides per animal) were evaluated. (**c**) Expression of the proximal tubular cell injury marker Kim1 and the dedifferentiation markers Pax2 and vimentin in the kidneys of sham and old mice treated with or without GDF11 at 72 h after IRI. Pictures are shown with 600 × magnification. (**d**) PCR for Pax2 and vimentin expression in the whole-kidney homogenates from old mice treated with or without GDF11 that underwent IRI. (**e**) Western blot for Kim1, Pax2, and vimentin expression in the whole-kidney homogenates from old mice treated with or without GDF11 that underwent IRI. Each lane represents a separate mouse. (**f**) Relative quantification of Kim1, Pax2, and vimentin expression as in (**e**), normalized to actin. For the percentage of EdU-positive tubular cells/HPF (**b**), values are means ± SD, n = 6 in each group. OIC, old mice that underwent IRI in the control group; OIG, old mice that underwent IRI in the GDF11 group. ^**^P < 0.01 vs. OIC. For analysis of Pax2 and vimentin mRNA levels (**d**), values are means ± SD, n = 6 in each group. OIC, old mice that underwent IRI in the control group; OIG, old mice that underwent IRI in the GDF11 group. ^##^*P* < 0.01 vs. Sham, ^**^P < 0.01 vs. OIC. For analysis of Kim1, Pax2, and vimentin protein levels (**f**), values are means ± SD, n = 3 in each group. OIC, old mice that underwent IRI in the control group; OIG, old mice that underwent IRI in the GDF11 group. ^#^*P* < 0.05 vs. Sham, ^##^*P* < 0.01 vs. Sham, ^**^P < 0.01 vs. OIC.

To further explore the role of GDF11 on IRI in old mice, we restored GDF11 in the kidneys of old mice by four i.p. injections of GDF11 (0.3 mg/kg/d) from 48 h before IRI to 48 h after IRI, and the control group was given an equal volume of vehicle. At 24 h and 72 h after IRI, renal function was assessed in the GDF11 group and control group. Although, there was no statistically significant difference in the increase of serum creatinine or BUN levels, or in the severity of tubular injury or necrotic tubules at 24 h after IRI, the time point of maximal renal injury in the GDF11 and control groups (Fig. 4a,b,d–f). The serum creatinine and BUN levels were significantly lower at 72 h after IRI in mice treated with GDF11 (Fig. 4a,b). Tubular injury in old mice treated with GDF11 was attenuated at 72 h after IRI (Fig. 4d,e). In fact, 30% of the surviving mice in the control group and 50% of the surviving mice in the GDF11 group survived at 72 h after IRI (Fig. 4c). Compared with controls, EdU-positive cells and LTL-positive cells, markers of newly divided cells and differential proximal tubular cells, respectively, were dramatically increased in the corticomedullary junction of kidneys in the GDF11 group. Over 20% of the proximal tubular cells were EdU-positive at 72 h after IRI, compared with only 10% EdU-positive proximal tubular cells in the control group (Fig. 5a,b). The Pax2 and vimentin levels were also upregulated in the kidneys of old mice treated with GDF11, while the Kim1 level was downregulated (Fig. 5c–f). GDF11 treatment increased injured tubular regeneration by accelerating tubular cell dedifferentiation and proliferation as well as improved kidney recovery and survival in old mice.

### Effect of GDF11 on proximal tubular cell dedifferentiation, proliferation, and migration *in vitro*

**Figure 6:**
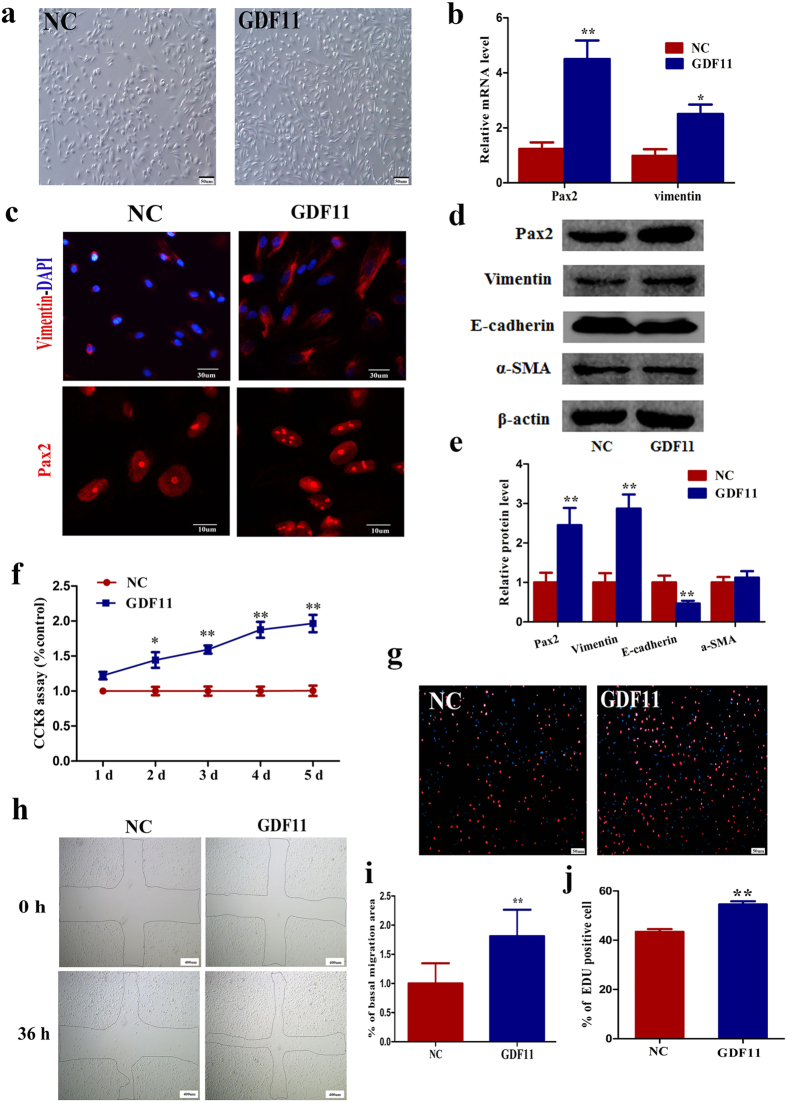
GDF11 increased proximal tubular cell dedifferentiation, proliferation, and migration *in vitro*. (**a**) HPTCs became elongated or stellate in shape, a feature of dedifferentiation, after rGDF11 exposure for 24 h. Magnification, 100×. (**b,e**) Effect of GDF11 on hPTC dedifferentiation. HPTCs were incubated with rGDF11 for 72 h, and then PCR, immunofluorescence, and western blotting were performed. (**b**) Relative mRNA levels of the dedifferentiation markers vimentin and Pax2. (**c**) Photographs of immunofluorescence staining for vimentin and Pax2. Magnification, 600 × for vimentin, and 1800 × for Pax2. (**d,e**) Relative protein levels of the dedifferentiation markers vimentin and Pax2, the epithelial marker E-cadherin, and the myofibrocyte marker α-SMA in hPTCs after GDF11 treatment for 72 h. (**f**) The number of hPTCs increased one fold after rGDF11 treatment for 5 days, as assessed by CCK8 tests. (**g,j**) The percentage of EdU-positive cells increased after rGDF11 incubation for 24 h. Representative images (**g**) and quantification (**j**) of the percentage of EdU-positive cells in hPTC cultures. Magnification, 100×. (**h,i**) Effect of GDF11 on migration following mechanical injury. Confluent monolayers of hPTCs were scraped to create mechanical injury and incubated for 36 h in the presence or absence of rGDF11 (80 ng/mL). (**h**) Photographs at 40 × magnification. (**i**) Migration into the wound area was quantified by measuring the migration of cells from the wound edge. For mRNA and protein analyses (**b,e**), data are means ± SD from three independent experiments. ^*^P < 0.05 vs. NC, ^**^P < 0.01 vs. NC. For the CCK8 assay (**f**), data are means ± SD from three independent experiments. ^*^P < 0.05 vs. NC, ^**^P < 0.01 vs. NC. For the EdU incorporation test (**j**), 15 LPFs (magnification,40×) per group from three independent experiments were evaluated. Data are means ± SD. ^**^P < 0.01 vs. NC. For measuring the migration of cells (**i**), data are means ± SD from three independent experiments (n = 3 in each group). ^**^P < 0.01 vs. NC.

To test whether GDF11 has a similar function on primary proximal tubular epithelial cells *in vitro*, normal hPTCs were treated with rGDF11 (80 ng/mL) or an equal volume of 0.1% BSA. HPTCs became elongated or stellate in shape, a feature of dedifferentiation, after GDF11 exposure for 24 h (Fig. 6a). Furthermore, rGDF11 treatment for 72 h also increased vimentin and Pax2 expression (Fig. 6b–e) and decreased E-cadherin expression, but did not affect the α-SMA level (Fig. 6d,e). After continuous treatment with rGDF11 for 5 days, the number of hPTCs significantly increased by one fold, according to the CCK8 assay (Fig. 6f). HPTCs were incubated with EdU for 24 h, and the number of EdU-positive cells in the GDF11 group increased 25%, compared to the control group (Fig. 6g,j). To elucidate the role of GDF11 on hPTC migration following injury, confluent hPTCs were mechanically injured and then incubated for 36 h. The hPTCs migrated into the denuded area and partially filled the wound area, which suggested that exogenous GDF11 enhanced wound closure (Fig. 6h,i). This finding indicated that GDF11 increased the dedifferentiation of primary proximal tubular epithelial cells and that these more-dedifferentiated cells had a significantly increased proliferation capacity and a higher migration capacity.

### The ERK1/2 signaling pathway was activated *in vitro* and *in vivo* after GDF11 treatment

**Figure 7:**
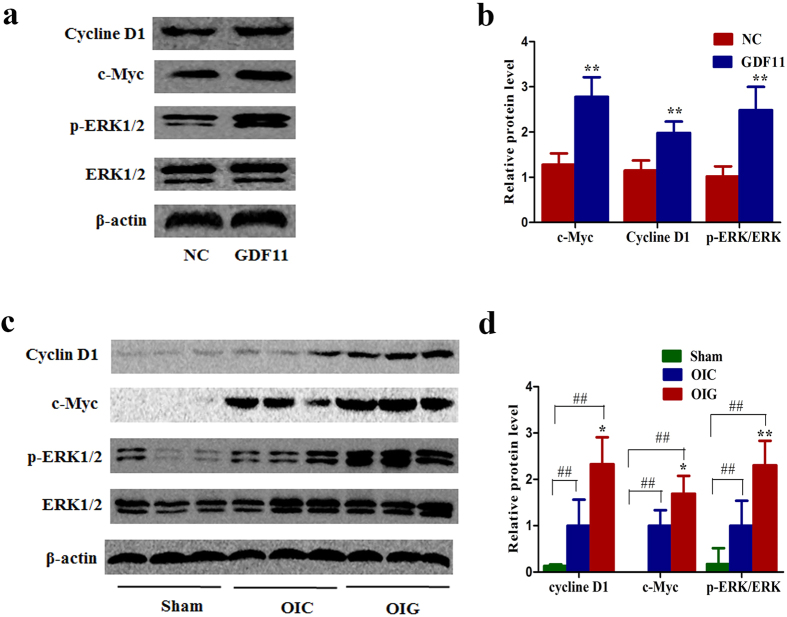
The ERK1/2 signaling pathway was activated *in vitro* and *in vivo* after GDF11 treatment. (**a,b**) ERK1/2 phosphorylation and the expression of its downstream effectors c-Myc and cyclin D1 were increased in hPTCs treated with rGDF11, as shown by western blot. (**c,d**) ERK1/2 phosphorylation and the expression of its downstream effectors c-Myc and cyclin D1 were increased at 72 h after IRI in the kidneys of old mice treated with GDF11. For protein analysis (**b**), data are means ± SD from three independent experiments. ^*^P < 0.05 vs. NC. For relative protein level determination (**d**), data are means ± SD; n = 3 in each group. OIC, old mice that underwent IRI for 28 min in the control group; OIG, old mice that underwent IRI for 28 min and were treated with GDF11. ^*^P < 0.05 vs. OIC, ^**^P < 0.01 vs. OIC. ^##^*P* < 0.01 vs. Sham.

GDF11 may share a common signaling pathway with the TGF-β/BMP superfamily. We used the TGF-β Pathway Phosphorylation Antibody Array to detect and analyze phosphorylation events at specific sites to identify GDF11 downstream effectors that regulate dedifferentiation and proliferation. We identified a spectrum of proteins whose phosphorylation levels were increased or decreased by more than 50% in two independent experiments. Many of these proteins are important for cell proliferation and migration when phosphorylated. These proteins included Abl1, c-Myc, ERK1/2, Smad1, and Akt (Table 1). We confirmed by immunoblotting that GDF11 administered to hPTCs resulted in increased ERK1/2 phosphorylation and activation levels (Fig. 7a,b). Meanwhile, GDF11 treatment also increased the expression of the ERK1/2 downstream effectors c-Myc and cyclin D1 in hPTCs (Fig. 7a,b). We further speculated whether activated ERK1/2 signaling also occurred at 72 h after IRI in the kidneys of old mice that underwent IRI and were treated with GDF11. We found that GDF11 administration increased the p-ERK1/2 level as well as cyclin D1 and c-Myc expression at 72 h after IRI in the kidneys of old mice that underwent IRI (Fig. 7c,d). These findings indicated that GDF11 could activate the ERK1/2 signaling pathway *in vitro* and *in vivo*.

### GDF11 regulates proximal tubular cell dedifferentiation and proliferation via an ERK1/2-dependent pathway

**Figure 8:**
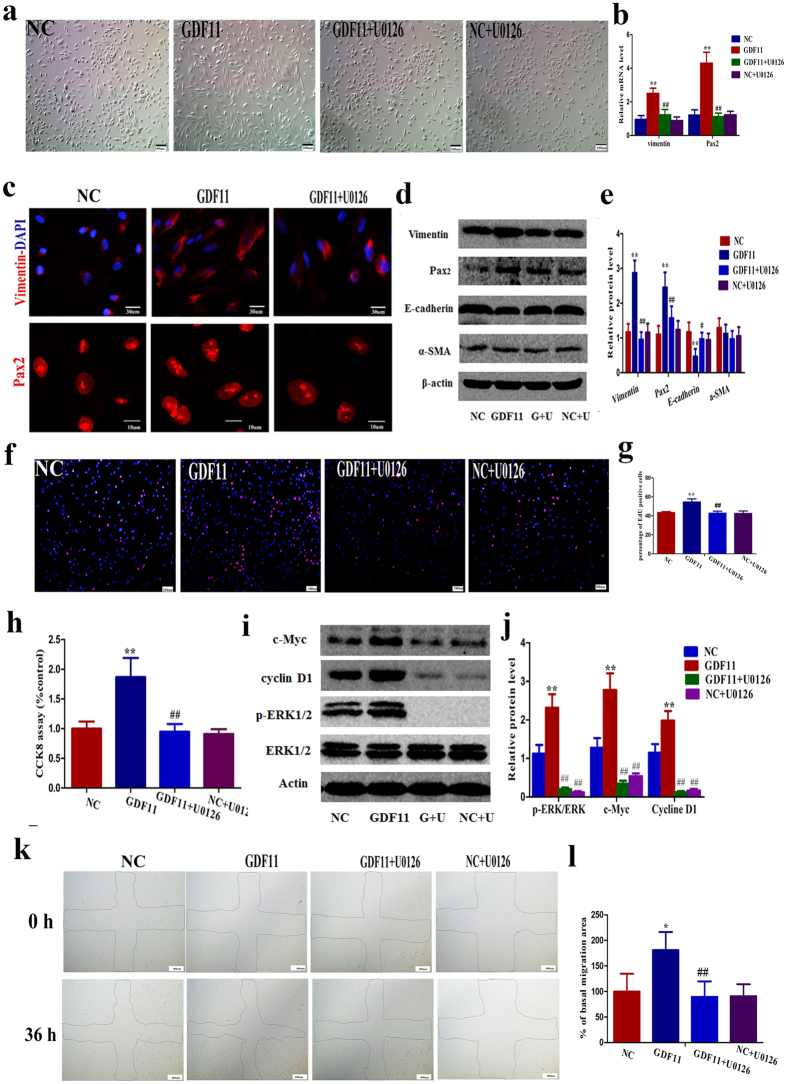
GDF11 increased proximal tubular cell dedifferentiation, proliferation, and migration via an ERK1/2-dependent pathway. (**a**) The morphological changes induced by GDF11 in hPTCs were inhibited by the ERK1/2 inhibitor U0126. Magnification,100×. (**b–e**) U0126 attenuated the increased hPTC dedifferentiation in the presence of GDF11. (**b**) mRNA levels for the dedifferentiation markers vimentin and Pax2. (**c**) Photographs of immunofluorescence staining for vimentin and Pax2. Magnification,600 × for vimentin and 1800 × for Pax2. (**d,e**) Protein levels of the dedifferentiation markers vimentin and Pax2, the epithelial marker E-cadherin, and the myofibrocyte marker α-SMA. (f–g) U0126 decreased the percentage of EdU-positive cells in hPTCs incubated with rGDF11 for 24 h. Magnification, 100×. (**h**) U0126 attenuated the increase in the number of hPTCs after GDF11 treatment for 5 days, as assessed by a CCK8 test. (**I,j**) The protein levels of the proliferation genes cyclin D1 and c-Myc were affected in hPTCs treated with either GDF11 or 0.1% BSA in the presence of U0126. (**k,l**) U0126 inhibited the effect of GDF11 on migration following mechanical injury. Confluent monolayers of hPTCs were scraped to create mechanical injury and incubated for 36 h in the presence of both GDF11 and U0126 or GDF11 alone. Photographs at 40 × magnification. For mRNA analysis (**b**), data are means ± SD from three independent experiments. ^**^P < 0.01 vs. NC, ^##^*P* < 0.01 vs. GDF11. For protein analysis (**e,j**), data are means ± SD from three independent experiments. ^**^P < 0.01 vs. NC, ^#^*P* < 0.05 vs. GDF11, ^##^*P* < 0.01 vs. GDF11. For the EdU incorporation test (**g**), 15 LPFs (magnification, 40×) per group from three independent experiments were evaluated. Data are means ± SD. ^**^P < 0.01 vs. NC, ^##^*P* < 0.01 vs. GDF11. For the CCK8 assay (**h**), data are means ± SD from three independent experiments. ^**^P < 0.01 vs. NC, ^##^*P* < 0.01 vs. GDF11. For measuring the migration of cells (**l**), data are means ± SD from three independent experiments (n = 3 in each group). ^*^P < 0.01 vs. NC, ^##^*P* < 0.01 vs. GDF11.

To verify the contribution of ERK1/2 on the effect of GDF11 on increasing dedifferentiation, proliferation, and migration, hPTCs were treated with rGDF11 or 0.1% BSA in the presence or absence of the ERK1/2 inhibitor U0126 (10 μM). U0126 weakened the GDF11-induced morphological changes (Fig. 8a) as well as the upregulation of the dedifferentiation markers Pax2 and vimentin and the downregulation of E-cadherin in the control group (Fig. 8b–e). U0126 did not affect hPTC morphology or Pax2, vimentin, and E-cadherin expression in the control group (Fig. 8b–e). U0126 inhibited the increase in proliferation in the presence of GDF11, without an influence in the control group (Fig. 8f–h). GDF11 attenuated the increased expression of the proliferation genes cyclin D1 and c-Myc in the presence of GDF11 (Fig. 8i,j). The GDF11-induced higher migratory capacity was blocked by U0126 (Fig. 8k,l). These results demonstrated that the effect of GDF11 on dedifferentiation as well as its relatively higher proliferation and migration were ERK1/2 dependent.

## Discussion

The GDF11 expression pattern in the circulation during aging has been a major unsolved question due to limitations in the detection technology^12^,^18^,^19^,^20^,^21^,^22^,^23^. Because the mature peptides of GDF11 and GDF8 are highly homologous, most anti-GDF11 antibodies, which target the C-terminus, cross-react with the GDF8 mature peptide. Unfortunately, the only GDF11-specific antibody (R&D Systems 743833), as reported by Egerman^12^, is not applicable for western blot because it cannot detect denatured protein. It binds to the protein epitope on the C-terminus of the GDF11 precursor. When it was used in ELISA, it detected mature GDF11, the C-cleavage product of the precursor, the prodomain-mature peptide latent complex, and the precursor, but it could not specifically detect the GDF11 mature peptide. The LC-MS/MS assay to quantify GDF11, reported recently, also could not specifically detect the GDF11 mature peptide^24^. Although we tried our best to find one specific antibody to detect the GDF11 mature peptide, we never succeeded.

Regarding the GDF11 level during organ senescence, Loffredo^10^ first reported that the mature GDF11 peptide (confirmed later to be GDF11/8) decreased with age in the spleen, and the expression of GDF11 mRNA in the kidney was very high, near that observed in the spleen. Then, Sinha reported that mature GDF11/8 peptide decreased with age in the skeletal muscle^11^. Later, Egerman declared that GDF11 mRNA expression was increased in the skeletal muscle of elderly mice. In our study, because no GDF8 mRNA was detected in the kidney, the GDF11 antibody ab124721 and orb101175 detected GDF11/8, most of which was GDF11. Therefore, we confirmed that the levels of GDF11 mRNA and precursor molecule increased with age and that the GDF11/8 (most of which was GDF11) mature peptide decreased with age in the kidney. The decrease of GDF11 mature peptide in the elderly kidney might be associated with PC5/6 activity, and the decline of GDF11 mature peptide might upregulate GDF11 mRNA and GDF11 precursor expression by feedback regulation.

The results regarding the function of GDF11 in regeneration after acute injury in the elderly also conflict. Sinha found that GDF11 improves muscle regeneration in old mice^11^, while Egerman contradicted this opinion and reported that GDF11 reduces skeletal muscle regeneration by inhibiting myoblast differentiation^12^. Tubular regeneration is distinct from skeletal muscle, whose regeneration after injury depends on the activity of mononuclear stem cells called satellite cells. The proliferating cells within the proximal tubule after AKI have been characterized either as dedifferentiated tubular epithelial cells, resident kidney stem cells, or even homed stem cells from outside the kidney. This latter possibility has been excluded. Humphreys used the Six2GFPCre driver line crossed to either Rosa26-LacZ or Z/Red reporter mice, resulting in heritable expression of either β-galactosidase (LacZ) or red fluorescent protein (RFP) in nearly all kidney epithelial cells (94–95%) following Cre-mediated recombination and no expression in extratubular cells. No dilution of the fate marker (β-Gal or RFP) was observed after AKI, which indicated that all repairable epithelial cells originated from within the tubule^25^. Very recently, Berger^5^ performed fate-tracing experiments of *scattered tubular cells* after IRI. On one hand, PECrtTA-labeled *scattered tubular cells* increase in number following injury, when cells were labeled after IRI. On the other hand, the frequency of labeled tubular cells remained constant, when cells were irreversibly labeled before IRI, indicating that the proliferating cells are definitively not a fixed intratubular progenitor population. Kusaba^6^ generated a knock-in mouse with a tamoxifen-inducible Cre recombinase (CreERt2) under the control of the sodium-dependent inorganic phosphate transporter SLC34a1 to perform fate tracing of fully differentiated epithelial cells. The bulk of labeled cells, which express CD24, CD133, vimentin, and Kim-1, markers of putative epithelial stem cells in the human kidney, proliferate after injury with an increased clone size after severe injury, compared with mild injury after injury and repair. When mice with completely labeled kidneys were subjected to injury and repair, there was no dilution of fate marker, despite substantial proliferation, indicating that unlabeled progenitors do not contribute to kidney repair. In conclusion, accumulating evidence has established that differentiated kidney epithelial cells, rather than scattered stem cells, dedifferentiate, proliferate, and repair injured tubules^5^,^6^,^25^.

Kidney regeneration after AKI is the common theme from the embryonic stage to the adult stage, and nephrogenic genes are reactivated as part of the process of kidney regeneration following AKI^26^. During metanephric development, GDF11 is expressed in the ureteric bud and the metanephric mesenchyme. Mice carrying a targeted deletion of *gdf11* possess a spectrum of renal abnormalities, with the majority of mutant animals lacking both kidneys^8^,^15^. However, there have been no reports on GDF8 expression in embryonic kidneys or adult kidneys, and GDF8-deficent mice have not been reported to have renal agenesis. Our studies confirmed that the GDF11/8 mature peptide (probably most of which was GDF11) in the kidney was upregulated after AKI. Dedifferentiation is a process by which differentiated tubular epithelial cells reverse the normal developmental processes and become mesenchymal-like progenitors expressing specific markers for progenitors in the metanephric mesenchyme, such as CD133, CD24, Pax2, and vimentin. These cells also exhibit an increased proliferation and migration capacity. Our study found that tubular cell dedifferentiation and proliferation were more attenuated in old mice after IRI and that supplementing GDF11 increased tubular cell dedifferentiation and proliferation in old mice that underwent IRI.

GDF11 induces phosphorylation and activation of the receptor regulated Smad (R-SMAD) proteins Smad2 and Smad3. Cellular responses to Smad2/3 activation are highly context dependent, and the presence or the absence of particular transcriptional cofactors, DNA-binding partners, and chromatin modifiers can dramatically alter the ultimate output of ligand binding^27^. GDF11 also signals through TGF-β noncanonical pathways, including ERK, JNK, and p38 MAPK^22^. In our study, GDF11 upregulated phosphorylation of Abl1, c-Myc, ERK1/2, Smad1, Smad2, and Akt in cultured hPTCs by the TGF-β Pathway Phosphorylation Antibody Array. Abl1, c-Myc, ERK1/2, and Akt are associated with cell proliferation. Smad2 protects against TGF-β/Smad3-mediated renal fibrosis^28^. Smad1 mediated the BMP7-Alk3 signaling pathway to antagonize epithelial–mesenchymal transition and favor mesenchymal–epithelial transition in unilateral ureteral obstruction, which is a well-established model of severe renal interstitial injury and fibrosis^29^.

In our study, GDF11 significantly activated ERK1/2 and increased the levels of the downstream factors cyclin D1 and c-Myc *in vitro* and *in vivo*. Blocking the ERK1/2 signaling pathway inhibited GDF11-induced primary tubular cell dedifferentiation, proliferation, and migration, which demonstrated that GDF11 induced primary tubular cell dedifferentiation though ERK1/2 signaling. ERK activation, which is required for cell proliferation, is implicated after AKI^30^,^31^. ERK1/2 activation has been shown to protect against oxidant, hypoxic, and ATP depletion-mediated injury of renal epithelial cells *in vitro*^32^, as well as oxidant and IRI *in vivo*^31^,^33^. Ras/Raf/ERK signaling also drives the dedifferentiation of Schwann cells upon nerve injury^34^, and ERK/c-Myc maintains both tumors and embryonic stem cells in an undifferentiated state, a fundamental early step in malignant transformation and cancer initiation^35^. In addition, GDF11 is abnormally highly expressed in renal cell cancer^16^ and colorectal cancer^36^, and its level is associated with high lymph node metastasis and cancer-related deaths^36^. GDF11 maintains erythroid precursors in a sustained dedifferentiation state and is a potential target to treat ineffective hematopoiesis in diseases such as myelodysplastic syndromes and β-thalassemia^37^,^38^.

Age is often associated with an increased onset and worse prognosis of AKI caused by a decrease in the adaption and regeneration capacity of tubular epithelial cells upon injury. However, because the timing of the ischemic insult is often unknown in human AKI, therapeutic efforts that accelerate tubular regeneration are highly practical for promoting organ recovery. Our data suggest that tubular cell dedifferentiation and proliferation were more attenuated in old mice after IRI, and supplementing GDF11 increased tubular cell dedifferentiation and proliferation as well as improved the prognosis of old mice that underwent IRI by upregulating the ERK1/2 signaling pathway. Our study focused on the effect of GDF11 on proximal tubular cell proliferation and tubular regeneration at 72 h after IRI. The effect of GDF11 on renal fibrosis after AKI needs to be explored 2 weeks after IRI in the future.

## Additional Information

**How to cite this article**: Zhang, Y. *et al*. GDF11 improves tubular regeneration after acute kidney injury in elderly mice. *Sci. Rep*. **6**, 34624; doi: 10.1038/srep34624 (2016).

## Supplementary Material

Supplementary Text

Supplementary Information

## Figures and Tables

**Table 1:** The ratio of each protein was determined as the ratio between the percentage of phosphorylated proteins to total proteins in GDF11 or 0.1% BSA (control) treatment.

Phosphorylation sites	Ratio 1 (GDF11/NC)	Ratio 2 (GDF11/NC)	Mean ratio (GDF11/NC)
Abl1 (Phospho-Thr754/735)	1.61	1.58	1.60
AKT (Phospho-Ser473)	2.03	1.31	1.67
ERK1-p44/42 (Phospho-Thr202)	2.61	2.66	2.64
Myc (Phospho-Ser62)	2.66	2.77	2.72
Smad1 (Phospho-Ser187)	2.16	2.11	2.14
Smad2 (Phospho-Ser250)	1.59	1.58	1.59
PKC theta (Phospho-Thr538)	0.34	0.11	0.23

Mean values from two independent experiments are shown.
