# Supplementary material for: GDF11 improves tubular regeneration after acute kidney injury in elderly mice
Source: Sci Rep. 2016 Oct 5;6:34624. doi: 10.1038/srep34624 (PMC5050408; doi:10.1038/srep34624)
Supplement: Supplementary Text [file srep34624-s1.doc]

**GDF11 improves tubular regeneration after acute kidney injury in elderly mice**

Ying Zhang^1,2^, Qinggang Li^1^_，_Dong Liu^1,3^, Qi Huang^1^, Guangyan Cai^1^, Shaoyuan Cui^1^, Xuefeng Sun^1^*, Xiangmei Chen^1^*

**Amino acid sequence of GDF11 and Anti-GDF11 antibodies List**

Amino acid sequence of GDF11

GDF11 protein NP_005802.1 human being

1mvlaaplllgflllalelrprgeaaegpaaaaaaaaaaaaagvggerssrpapsvapepd

61gcpvcvwrqhsrelrlesiksqilsklrlkeapnisrevvkqllpkapplqqildlhdfq

121gdalqpedfleedeyhattetvismaqetdpavqtdgsplcchfhfspkvmftkvlkaql

181wvylrpvprpatvylqilrlkpltgegtaggggggrrhirirslkielhsrsghwqsidf

241kqvlhswfrqpqsnwgieinafdpsgtdlavtslgpgaeglhpfmelrvlentkrsrrnl

301gldcdehssesrccrypltvdfeafgwdwiiapkrykanycsgqceymfmqkyphthlvq

361qanprgsagpcctptkmspinmlyfndkqqiiygkipgmvvdrcgcs

GDF11 mature peptide: Asn299-Ser407

Anti-GDF11 antibodies List

| Anti-GDF11 antibody | Immunogen | Application | Specify | Reactivity | Host |
| --- | --- | --- | --- | --- | --- |
| R&D  743833 | Asn299-Ser407 | IHC ELISA | GDF11 | human | Mouse |
| Abcam  Ab 124721 | Met350-Ser407 | WB IHC | GDF11/8 | human mouse rat et al | Rabbit |
| Biorbyt  Orb 101175 | ASn292-GLu306 | WB ELISA | GDF11/8 mature peptide | human mouse | Rabbit |
| LifeSpan BioSciences  LS-C138772 | Met350-Val400 | WB ELISA | GDF11/8 | Human mouse rat | Rabbit |
