# Supplementary material for: GDF11 improves tubular regeneration after acute kidney injury in elderly mice
Source: Sci Rep. 2016 Oct 5;6:34624. doi: 10.1038/srep34624 (PMC5050408; doi:10.1038/srep34624)
Supplement: Supplementary Information [file srep34624-s2.doc]

**GDF11 improves tubular regeneration after acute kidney injury in elderly mice**

Ying Zhang^1,2^, Qinggang Li^1^_，_Dong Liu^1,3^, Qi Huang^1^, Guangyan Cai^1^, Shaoyuan Cui^1^, Xuefeng Sun^1^*, Xiangmei Chen^1^*


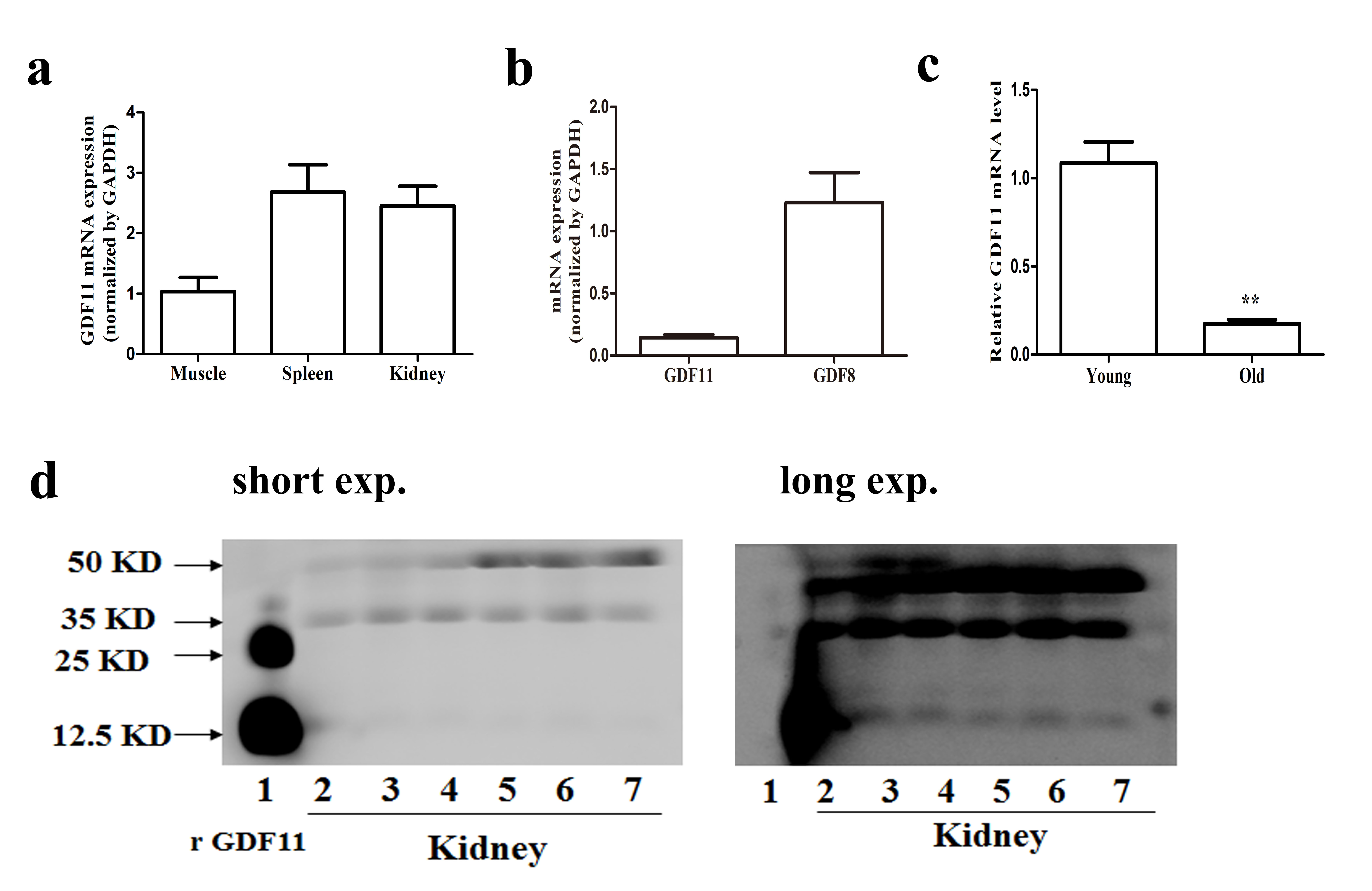


Supplementary Figure GDF11 mRNA and protein expressed in the spleen, muscle, and kidney in 3-month mice and 24-month mice. (a) Relative GDF11 mRNA level in the muscle, spleen and kidney in 3-month (young) mice. Values are means± SD, n=6 in each group. (b) Relative GDF11 and GDF8 mRNA level in the skeletal muscle of 3-month (young) mice. Values are means± SD, n=6 in each group. (c) Relative GDF11 mRNA expressed in the spleen of young mice and old mice. Values are means± SD, n=6 in each group, ^**^*P*<0.01 vs. young. (d) Western blot detected GDF11 protein expression in the kidneys of young mice aged 3 months at 50 kDa for the GDF11 precursor, 35 kDa for the C-cleavage precursor product, and 12.5 kDa for the mature peptide. The 25 kDa band is for the rGDF11 dimer, and the 12.5 kDa band is for the rGDF11 monomer. Short exp., short exposure. Long exp., long exposure after covering the rGDF11 band.

Table S1, The serum creatinine of young mice under IRI for 28min/35min/40min and old mice under IRI for 28min/35min at IRI 24h.

|  | Young | Old | *P* 值 |
| --- | --- | --- | --- |
| Sham | 0.16±0.04 | 0.18±0.05 | 0.560 |
| IRI-28min | 0.97±0.16 | 1.70±0.23 | 0.000 |
| IRI-35min | 1.79±0.18 | 2.18±0.29 | 0.035 |
| IRI-40min | 2.23±0.45 |  |  |

Table S1, The serum creatinine level (mg/dl) of young mice under IRI for 28min/35min/40min and old mice under IRI for 28min/35min at IRI 24h. N
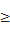
6 in each group. The data were shown by mean±SD.

Table S2, The serum BUN level of young mice under IRI for 28min/35min/40min and old mice under IRI for 28min/35min at IRI 24h.

|  | Young | Old | *P* 值 |
| --- | --- | --- | --- |
| Sham | 20.49±5.62 | 41.06±5.06 | 0.000 |
| IRI-28min | 88.4±22.1 | 228.6±48.2 | 0.000 |
| IRI-35min | 155.85±40.04 | 266.4±52.3 | 0.042 |
| IRI-40min | 320.5±51.4 |  |  |

Table S2, The serum BUN level (mg/dl) of young mice under IRI for 28min/35min/40min and old mice under IRI for 28min/35min at IRI 24h. N
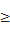
6 in each group. The data were shown by mean±SD.

Table S3, The percentage of necrotic tubules of young mice under IRI for 28min/35min/40min and old mice under IRI for 28min/35min at IRI 24h.

|  | Young | Old | P 值 |
| --- | --- | --- | --- |
| Sham | — | — |  |
| IRI-28min | 30.6±8.2 | 48.5±8.6 | 0.040 |
| IRI-35min | 45.6±10.3 | 68.8±13.2 | 0.035 |
| IRI-40min | 62.5±16.4 |  |  |

Table S3, The percentage of necrotic tubules of young mice under IRI for 28min/35min/40min and old mice under IRI for 28min/35min at IRI 24h. Approximately 40 high-power fields (HPFs,
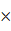
400) per individual mouse (10 HPFs per slide, four slides per animal) were evaluated. n = 6 in each group. The data were shown by mean±SD.
